# A Reproducible Mouse Model of Moderate CKD With Early Manifestations of Osteoblastic Transition of Cardiovascular System

**DOI:** 10.3389/fphys.2022.897179

**Published:** 2022-04-29

**Authors:** Sarah E Machado, Daryll Spangler, Laurence M. Black, Amie M. Traylor, József Balla, Abolfazl Zarjou

**Affiliations:** ^1^ Division of Nephrology, Department of Medicine, University of Alabama at Birmingham, Birmingham, Hungary; ^2^ ELKH-UD Vascular Biology and Myocardial Pathophysiology Research Group, Division of Nephrology, Department of Medicine, Faculty of Medicine, Hungarian Academy of Sciences, University of Debrecen, Debrecen, Hungary

**Keywords:** chronic kidney disease, vascular calcification, ischemia, reperfusion, mouse model, cardiac calcinosis

## Abstract

Chronic kidney disease (CKD) is a significant public health challenge with a substantial associated risk of mortality, morbidity, and health care expenditure. Culprits that lead to development and progression of CKD are multifaceted and heterogenous in nature. This notion underscores the need for diversification of animal models to investigate its pathophysiology, related complications, and to subsequently enable discovery of novel therapeutics. Importantly, animal models that could recapitulate complications of CKD in both genders are desperately needed. Cardiovascular disease is the most common cause of death in CKD patients that may be due in part to high prevalence of vascular calcification (VC). Using DBA/2 mice that are susceptible to development of VC, we sought to investigate the feasibility and reproducibility of a unilateral ischemia-reperfusion model followed by contralateral nephrectomy (UIRI/Nx) to induce CKD and its related complications in female and male mice. Our results demonstrate that irrespective of gender, mice faithfully displayed complications of moderate CKD following UIRI/Nx as evidenced by significant rise in serum creatinine, albuminuria, higher degree of collagen deposition, elevated expression of classic fibrotic markers, higher circulating levels of FGF-23, PTH and hepcidin. Moreover, we corroborate the osteoblastic transition of aortic smooth muscle cells and cardiomyocytes based on higher levels of osteoblastic markers namely, Cbfa-1, osteopontin, osteocalcin, and osterix. Our data confirms a viable, and consistent model of moderate CKD and its associated complications in both male and female mice. Furthermore, early evidence of osteoblastic transition of cardiovascular system in this model confirms its suitability for studying and implementing potential preventive and/or therapeutic approaches that are urgently needed in this field.

## Introduction

Chronic kidney disease (CKD) is a worldwide public health problem affecting ∼850 million people including 37 million Americans (Center for disease Control, 2021). Advanced CKD is associated with several adverse clinical outcomes, such as accelerated cardiovascular diseases, kidney failure requiring kidney replacement therapy, mineral bone disease, anemia, metabolic and endocrine abnormalities, and poor quality of life (Couser et al., 2011; Bello et al., 2017). The substantial burden of CKD has been recognized by the recent “Advancing American Kidney Health (AAKH)” plan through an Executive Order in July 2019 (U.S. Department of Health and Human Services, 2019). Two of the major goals of the AAKH initiative are to slow progression of CKD and delay the need for kidney replacement therapy. To achieve these objectives animal models that could faithfully recapitulate human CKD complications in both males and female animals are essential.

Cardiovascular disease is the main cause of mortality in CKD patients that may in part be related to higher incidence of vascular calcification (VC) which is a hallmark of advanced CKD and end stage kidney disease (ESKD) (Muntner et al., 2002; Manjunath et al., 2003; Briasoulis and Bakris, 2013; Manzoor et al., 2018). Importantly, VC is an independent risk factor for morbidity and mortality beyond established risk factors (London et al., 2003; Fox et al., 2004; Haydar et al., 2004; Sigrist et al., 2007). This premise is further supported by presence of VC in young patients with advanced CKD (Braun et al., 1996; Goodman et al., 2000). Such calcification is an active process where smooth muscle cells (SMCs) at the level of tunica media gain an “osteoblast” phenotype through upregulated expression of several osteogenic genes and markers. (Giachelli, 2004; Cozzolino et al., 2006; O'Neill, 2017; Vervloet and Cozzolino, 2017; Dube et al., 2021). Moreover, this process of osteoblastic transition and calcium deposition also extends to cardiomyocytes and valvular tissue leading to cardiac calcinosis and valvular mineralization (Rostand et al., 1988; Hujairi et al., 2004; Kempf et al., 2009; Urena-Torres et al., 2020). VC results in multiple hemodynamic derangements that include loss of arterial elasticity, increase in pulse wave velocity, development of left ventricular hypertrophy and decrease in coronary artery perfusion ensuing in myocardial ischemia and failure (Mizobuchi et al., 2009; Zhu et al., 2012).

Many factors have been described that accelerate the osteoblastic transition process and ultimately lead to an imbalance between pro- and anti-mineralization processes. These include disorders of calcium and phosphate metabolism, reactive oxygen species, apoptosis, elastin degradation, defective DNA damage response, iron and magnesium homeostasis, cellular senescence, upregulation of pro-mineralization microRNAs, and downregulation of endogenous inhibitors of ectopic calcification such as fetuin-a, matrix gla protein (MGP) and inorganic pyrophosphate (Davies and Hruska, 2001; Moe and Chen, 2008; O'Neill et al., 2011; Fakhry et al., 2018; Villa-Bellosta and O'Neill, 2018; Balla et al., 2019; Ciceri and Cozzolino, 2021). Despite recognition and vigorous investigations, there is no specific therapy to mitigate progression and/or revert VC. Furthermore, it is evident that extensive mineralization of cardiovascular system is an irreversible process. Hence, investigation of potential therapeutic approaches requires an animal model where interventions could be utilized and tested during early stages of osteoblastic transition of cardiovascular cells and calcification of the vascular tree.

Establishing an animal model of CKD ought to account for various elements that govern the reliability of such model. These factors include 1) establishment of CKD within a practical time frame to minimize housing and financial burden, 2) mimic pathological aspects of human CKD, 3) establish CKD in both male and female mice to enable meaningful translational efforts as directed by the National Institutes of Health (Clayton and Collins, 2014).

Here, using DBA/2 mice that unlike commonly used C57BL/6 mice are known to be susceptible to VC (van den Broek et al., 1998; Brunnert et al., 1999), we introduce a mouse model that manifests prominent characteristics of moderate CKD in both genders, and osteoblastic transition of cardiovascular cells.

## Materials and Methods

### Animals and Diet Details

Male and female DBA/2 mice (stock number 000671) were purchased from Jackson Laboratories (Jackson Labs, Bar Harbor, ME) and maintained in a sterile, controlled environment. All procedures involving mice were performed in accordance with National Institutes of Health guidelines regarding the care and use of live animals and were reviewed and approved by the Institutional Animal Care and Use Committee of University of Alabama at Birmingham. During the unilateral ischemia/reperfusion (UIRI) studies all animals (sham and experimental groups) were maintained on high phosphate diet starting at 3 days after the second surgery. The high phosphate diet was formulated to contain 0.9% phosphate and 0.6% calcium and was purchased from Dyets Inc. (Bethleham, PA). Mice were harvested at the end of 16 weeks to assess kidney function and collect tissues. The adenine diet used to induce CKD and VC was formulated to contain 0.2% adenine, 0.6% Ca, and 2% phosphate (diet manufactured by Envigo, Madison, WI). Control mice received 0.6% Ca and 0.9% phosphate without adenine supplementation. Mice were harvested at the end of 4 weeks and only female mice were used in these experiments.

### Surgical Procedure

CKD was induced in 8–12 weeks old mice by way of a two-step UIRI surgical and contralateral nephrectomy procedure. A total of 12 female and six male mice were used for these studies. During surgery 1, the right kidney was exposed, and ischemia induced by clamping the renal pedicle with an 86 g micro-serrefine (Fine Science Tools, Foster City, CA) for 27–30 min. Kidneys were inspected for color change within 1 min of clamp removal to ensure uniform reperfusion. Surgery two was performed following a 1-week recovery period and consisted of a total-left nephrectomy. Data presented here include both 27 and 30 min UIRI as there was no statistically significant difference in creatinine or observance of differing collagen deposition between the models. Sham operated animals underwent a surgical procedure with dorsal incisions made and the kidney surfaced and placed back in the abdominal cavity. At 72 h post-recovery, both sham and experimental groups of mice were maintained on a high phosphate diet and sacrificed at 16 weeks. During both procedures, mice were anesthetized using intraperitoneally injected ketamine/xylazine with depth of anesthesia determined by toe-pinch reflex and body temperature maintained at 36.5 ± 0.5°C. Additionally, mice were administered buprenorphine (0.05–0.1 mg/kg) immediately pre-op and 12 h post op as analgesic. Mice were allowed free access to food and water before and after surgery. All surgical procedures were carried out by the UAB O’Brien Center (UAB-UCSD O’Brien Center Microsurgical Core, Birmingham, AL P30 DK079337).

### Western Blot Analysis

Harvested tissues were homogenized in RIPA buffer (50 mmol/L Tris-HCl, 1% NP-40, 0.25% deoxycholic acid, 150 mmol/L NaCl, 1 mmol/L EGTA, 1 mmol/L sodium orthovanadate, and 1 mmol/L sodium fluoride) with the addition of protease inhibitor (Sigma-Aldrich, St. Louis, MO) and phosphatase inhibitor cocktail (Bimake, Houston, TX). Lysates were spun at 12,000 RPM for 15 min at 4°C and supernatant was collected. Total protein was quantified by BCA protein assay (Thermo Scientific, Waltham, MA) and loaded on a 12% Tris-glycine sodium dodecyl sulfate polyacrylamide electrophoresis gel at a concentration of 40 µg and transferred to an Immobilon-P PVDF membrane via electroblotting (Millipore-Sigma, St. Louis, MO). Following transfer, membranes were incubated in 6% non-fat dry milk in PBS with 0.1% Tween-20 (Fisher Scientific, Suwanee, GA) for 1 h at room temperature and incubated overnight at 4°C overnight in 1–5% non-fat dry milk with mouse osteopontin (Santa Cruz Biotechnologies, Dallas, TX, 1:250), mouse osteocalcin (Santa Cruz Biotechnologies, 1:250), mouse Cbfa-1 (Santa Cruz Biotechnologies, 1:250), and mouse GAPDH (Millipore-Sigma, 1:10,000). Membranes were incubated for 1 h at room temp with HRP-conjugated anti-mouse or anti-rabbit secondary antibodies (Kindle Biosciences LLC, US). Membranes were imaged for peroxidase activity of target proteins using a KwikQuant digital imaging system with enhanced chemi-luminescence substrate (Kindle Biosciences LLC). Analysis of densitometry was performed using Licor Image Studio Lite (version 5.2) and normalized to GAPDH expression.

### Serum Chemistries

Blood was collected from anesthetized mice maintained at 2% isoflurane via cardiac puncture and collected in serum separation tubes incubated for 30 min at room temperature. Serum was isolated by centrifugation and subsequent chemistry analysis performed using enzyme-linked immunosorbent assays. Serum intact PTH 1-84 (Quidel Corporation, San Diego, CA) and hepcidin (Elabscience, Houston, TX), levels were measured using a BioTek Synergy HTX Multi-Mode Reader (BioTek Instruments, Inc., Highland Park, MO) at wavelengths specified by manufacturer protocol. Serum phosphate and iron levels were analyzed by the Animal Histopathology and Laboratory Medicine Core, University of North Carolina-Chapel Hill, Chapel Hill, NC. Serum creatinine levels were performed by the UAB O’Brien Center using liquid chromatography-tandem mass spectrometry (UAB-UCSD O’Brien Center Bioanalytical Resource Core, Birmingham, AL).

### Picrosirius Red Stain

Picrosirius red (PSR) staining was performed as previously described (Zarjou et al., 2011c). Briefly, kidneys were fixed in 10% neutral buffered formalin for 24 h then embedded in paraffin. Collagen deposition was visualized as described previously using PSR. Sections were deparaffinized in xylene washes and rehydrated, incubated in PSR for 1 h, washed in acidified water, and dehydrated. Sections were mounted in a resinous medium. All images were acquired on a BZ-X700 All-In-One Fluorescence Microscope (Keyence, Istasca, IL). Images were acquired using a 20X magnification.

### Alizarin Red Stain

Alizarin red stain to visualize calcium deposition on heart and aorta sections were performed as previously described (Zarjou et al., 2011b). Briefly, heart and aorta sections were prepared in the same aforementioned manner as kidney sections prior to paraffin embedding and sectioning. Sections were stained with 2% alizarin red (Sigma-Aldrich, St. Louis, MO) pH 4.3 in di H_2_O for 15–20 min rinsed twice in PBS. Slides were dehydrated and cleared in xylenes with mounting procedures mentioned above. Slides were imaged using a Keyence BZ-X700 microscope. Images were obtained using a 4X magnification. To visualize abdominal aortae calcium deposition alizarin red staining was performed as previously described (Leroux-Berger et al., 2011). The abdominal aortae were dissected between the diaphragm and common illiac arteries and fixed for 24 h at room temperature in 95% ethanol and then directly stained with alizarin red in 1% KOH for 24 h, rinsed in 2% KOH, and conserved in a solution containing 50% glycerol.

### Immunohistochemistry

Immunohistochemistry was performed as previously described (Zarjou et al., 2013). Briefly, kidneys were fixed in 10% neutral buffered formalin for 24 h and then embedded in paraffin. Paraffin-embedded 5-μm kidney sections were deparaffanized in xylenes, rehydrated in a series of ethanol rinses from 100% to 70% ethanol, then washed in distilled water. Antigen retrieval was performed in Trilogy (Cell marque) at 95°C for 30 min. Sections were allowed to cool slowly, washed in distilled water, and incubated in 3% H_2_O_2_ for 20 min. Sections were blocked in blocking buffer containing 5% goat serum in PBS, 0.1% Tween-20 (PBST), at room temperature for 1 h. Primary antibodies were diluted in the blocking buffer for rabbit fibronectin (Millipore-Sigma, 1:250), rabbit osterix (Abcam, 1:250) and added to sections overnight at 4°C. Sections were washed 3 times with PBST for 5 min each. Goat anti-rabbit secondary antibody (Jackson ImmunoResearch Laboratories; 1:500) was diluted in blocking buffer and added to the sections for 1 h at room temperature. Sections were washed 3 times with PBST for 5 min each. Chromagen substrates were mixed per the manufacturer’s instructions (Vector Labs) and added to sections. Sections were washed in distilled water, dehydrated, and mounted using xylene mounting media (PROTOCOL).

### Determination of Urinary Albumin

Urinary albumin was quantified using the Mouse Albumin ELISA kit (Bethyl Laboratories, Montgomery, TX), and data are presented as an albumin-to-creatinine ratio. The kit components were reconstituted, and the assay was performed according to the manufacturer’s instruction.

### Determination of Serum FGF-23 Levels

Serum FGF-23 was quantified using mouse FGF-23 ELISA kit (Abcam, Waltham, MA). The kit components were reconstituted, and the assay was performed according to the manufacturer’s instruction. Data are presented as picogram/milliliter.

### Statistical Analysis

Data are presented as mean ±SEM with individual data points and no data points were excluded from the analysis. Statistical analyses were performed using GraphPad Prism (version 9, San Diego, CA). ANOVA and Tukey’s multiple comparisons post hoc test were performed for comparisons between groups. Two-group comparisons were made using unpaired Student’s t-tests. The value of *p* < 0.05 was considered significant.

## Results

### Establishing CKD via Unilateral Ischemia-Reperfusion Followed by Contralateral Nephrectomy

**FIGURE 1:**
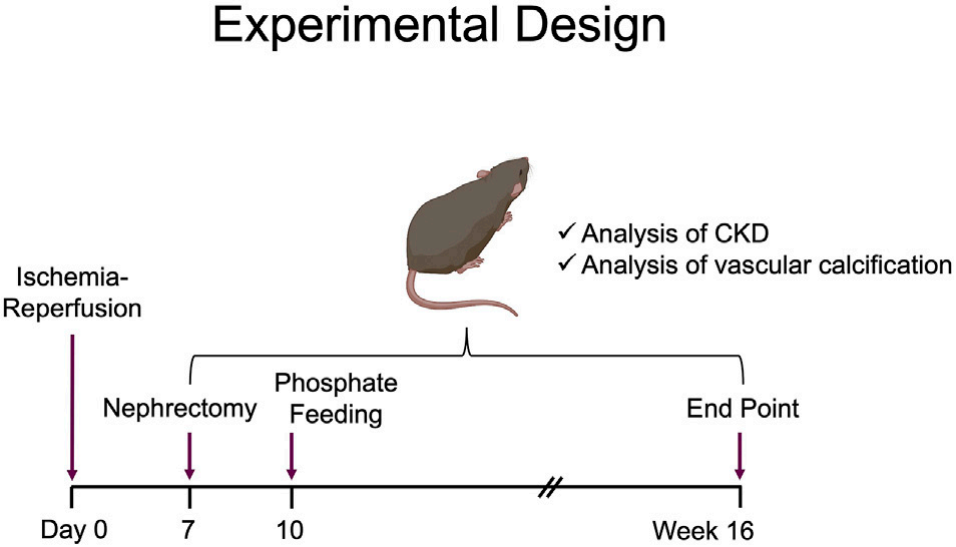
Schematic presentation of overall experimental design.

**FIGURE 2:**
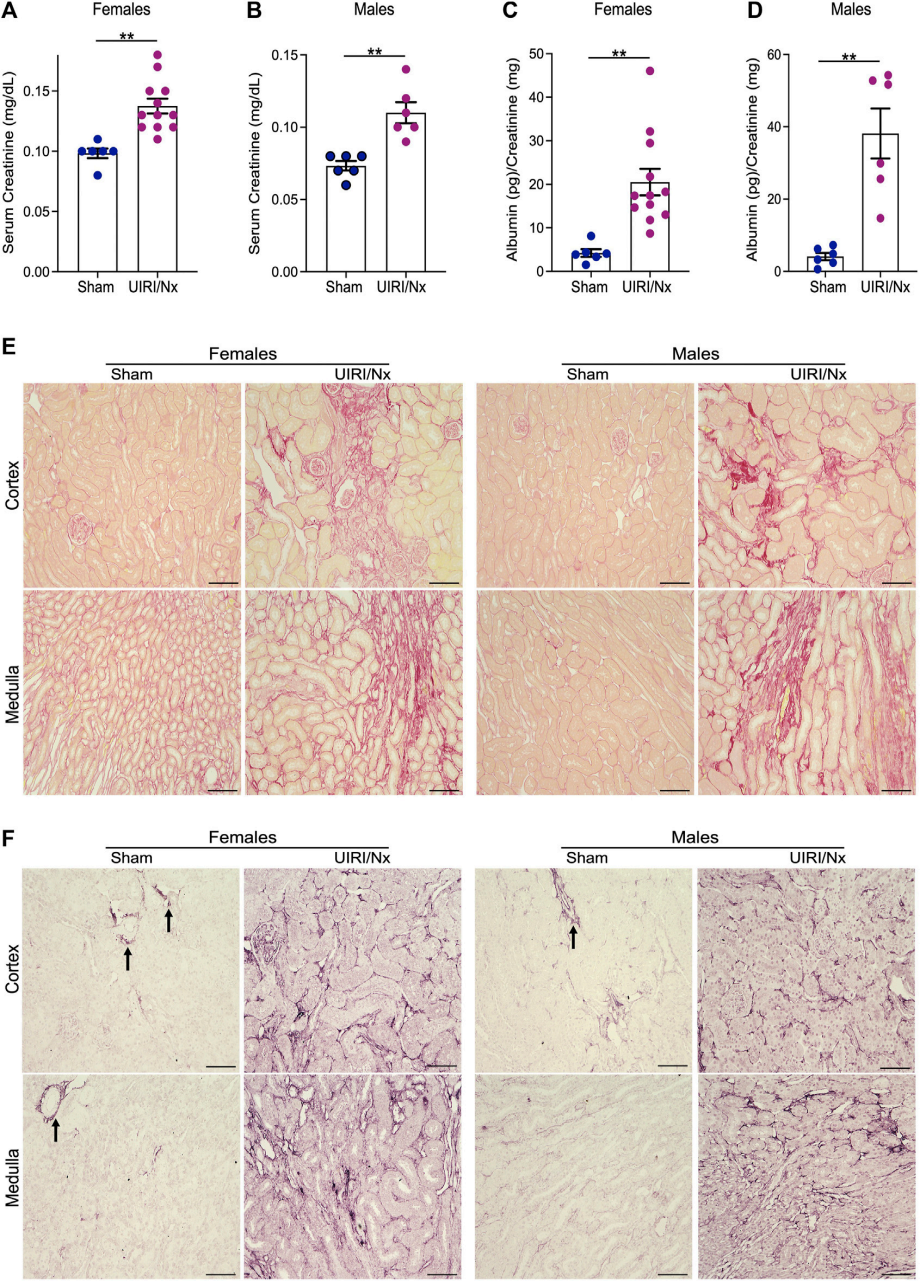
Establishment of CKD and via unilateral ischemia-reperfusion followed by contralateral nephrectomy. **(A,B)** Serum creatinine levels were measured and expressed as milligrams/deciliter in sham and experimental group of mice 16 weeks post contralateral nephrectomy. Females are represented in (panel A) and males in (panel B). Data are expressed as means ± SEM; **p < 0.01 vs. Sham. **(C,D)** Urine was collected at the time of sacrifice via bladder puncture and evaluated for levels of albumin. Results were then normalized to urine creatinine levels and expressed as picogram per milligram for females (panel C) and males (panel D). Data are expressed as means ± SEM; **p < 0.01 vs. Sham. n = 6/sham female and male groups. n = 12/female UIRI/Nx group and n = 6/male UIRI/Nx group. **(E)** Picrosirius red stain demonstrating collagen deposition validates increased collagen in both genders that belong to UIRI/Nx group compared to their sham operated littermates. Images are representative of six independent experiments. Scale bar = 100 μm. **(F)** kidney sections were prepared, and immunohistochemistry assays were performed using anti-fibronectin to determine the pattern of expression of these proteins. Arrows indicate positive fibronectin signal in sham operated mice around the arterioles. Images are representative of six independent experiments. Scale bar = 100 μm. UIRI/Nx = unilateral ischemia/reperfusion followed by contralateral nephrectomy.

The experimental design is illustrated in Figure 1. To induce CKD, mice were subjected to UIRI mediated kidney injury, followed by contralateral nephrectomy 7 days post UIRI. Three days post nephrectomy both sham and experimental groups were placed on high phosphate diet (0.9%) for 16 weeks. Sham group of mice were also placed on high phosphate diet to confirm the additive effect of CKD given susceptibility of DBA/2 mice to VC. All mice survived following both surgeries and no mortalities were observed throughout the study. To verify kidney injury, serum creatinine was measured via retro-orbital bleed 1 day post contralateral nephrectomy which showed consistently elevated serum creatinine levels. Males had average serum creatinine of 0.86 mg/dl, SEM ±0.18 and we found females to have average serum creatinine of 0.4 mg/dl, SEM ±0.03. We also measured food consumption and weight changes on a weekly basis and did not observe any differences between the two groups. At 16 weeks, serum creatinine measurements corroborated reduced renal function and hence establishment of CKD. We demonstrate that serum creatinine was significantly higher in both females (Figure 2A) and males (Figure 2B) who underwent UIRI/Nx compared to sham operated littermates. To investigate another characteristic finding of CKD, we assessed degree of proteinuria by measuring urinary albumin levels and found that irrespective of sex UIRI/Nx resulted in significant degree of albuminuria (Figure 2C,D). Interstitial fibrosis is a hallmark of CKD and we accordingly utilized picrosirius stain to visualize degree of collagen deposition. Our data illustrates that both genders in UIRI/Nx group displayed markedly higher levels of collagen deposition compared to sham operated animals (Figure 2E). To confirm these findings, we also investigated protein expression levels of a classic fibrotic marker, fibronectin. As expected, both female and male mice that underwent sham surgery only demonstrated fibronectin expression around the tunica adventitia of arterioles (Figure 2F). In contrast both genders displayed markedly higher tubular and interstitial levels of fibronectin when subjected to UIRI/Nx (Figure 2F).

### Validation of CKD Establishment and Related Complications via Elevated FGF-23, Parathyroid Hormone and Hepcidin

**FIGURE 3:**
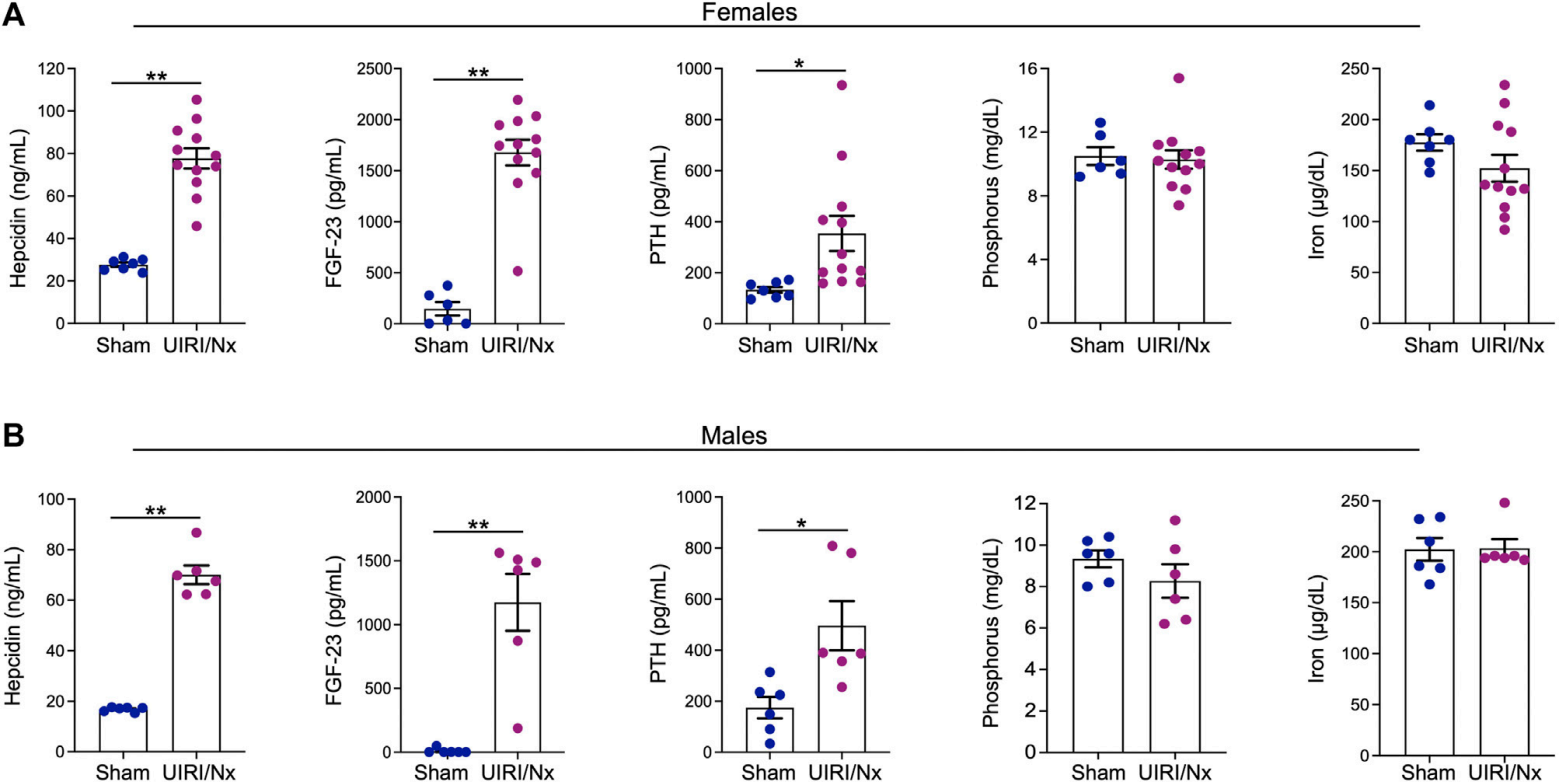
UIRI/Nx model is consistently associated with secondary hyperparathyroidism, elevated FGF-23, and hepcidin levels. At 16 weeks post contralateral nephrectomy, serum levels of hepcidin, FGF23, parathyroid hormone (PTH), phosphorus, and iron levels were quantified in both females **(A)** and males **(B)**. Data are expressed as mean ± SEM. *p < 0.05 vs. Sham and **p < 0.01 vs. Sham. n = 6/sham female and male groups. n = 12/female UIRI/Nx group and n = 6/male UIRI/Nx group.

CKD often translates to disruptions in multiple endocrine, electrolyte and mineral pathways. Hepcidin, an acute phase reactant primarily produced by hepatocytes and excreted by the kidneys, is the fundamental regulator of systemic iron metabolism (Zarjou et al., 2011a). Circulating hepcidin levels are commonly elevated in patients with CKD that may be the consequence of decreased renal elimination and increased production given a chronic inflammatory milieu (Zarjou et al., 2011a). Here, we show that serum hepcidin levels were significantly higher in both females (Figure 3A) and males (Figure 3B) in UIRI/Nx group when compared to sham operated littermates. CKD is also often associated with increments in circulating levels of fibroblast growth factor-23 (FGF-23) and parathyroid hormone (PTH). Correspondingly, we measured circulating levels of both proteins and demonstrate that both FGF-23 and PTH levels were significantly increased in the experimental group compared to the sham group in both females and males (Figure 3A,B). It is noteworthy that significant rise in serum PTH levels are generally first seen in patients with CKD IIIb-IV (Levin et al., 2007). In contrast we did not find any distinct differences in serum phosphate and serum iron levels between the two groups (Figure 3A,B). This finding also corroborates human CKD population where hyperphosphatemia and iron deficiency are generally a complication of very late stages of CKD and ESKD (Levin et al., 2007) where the presented model embodies early and moderate CKD.

### Osteoblastic Transition of Cardiovascular System Following UIRI/Nx and High Phosphate

**FIGURE 4:**
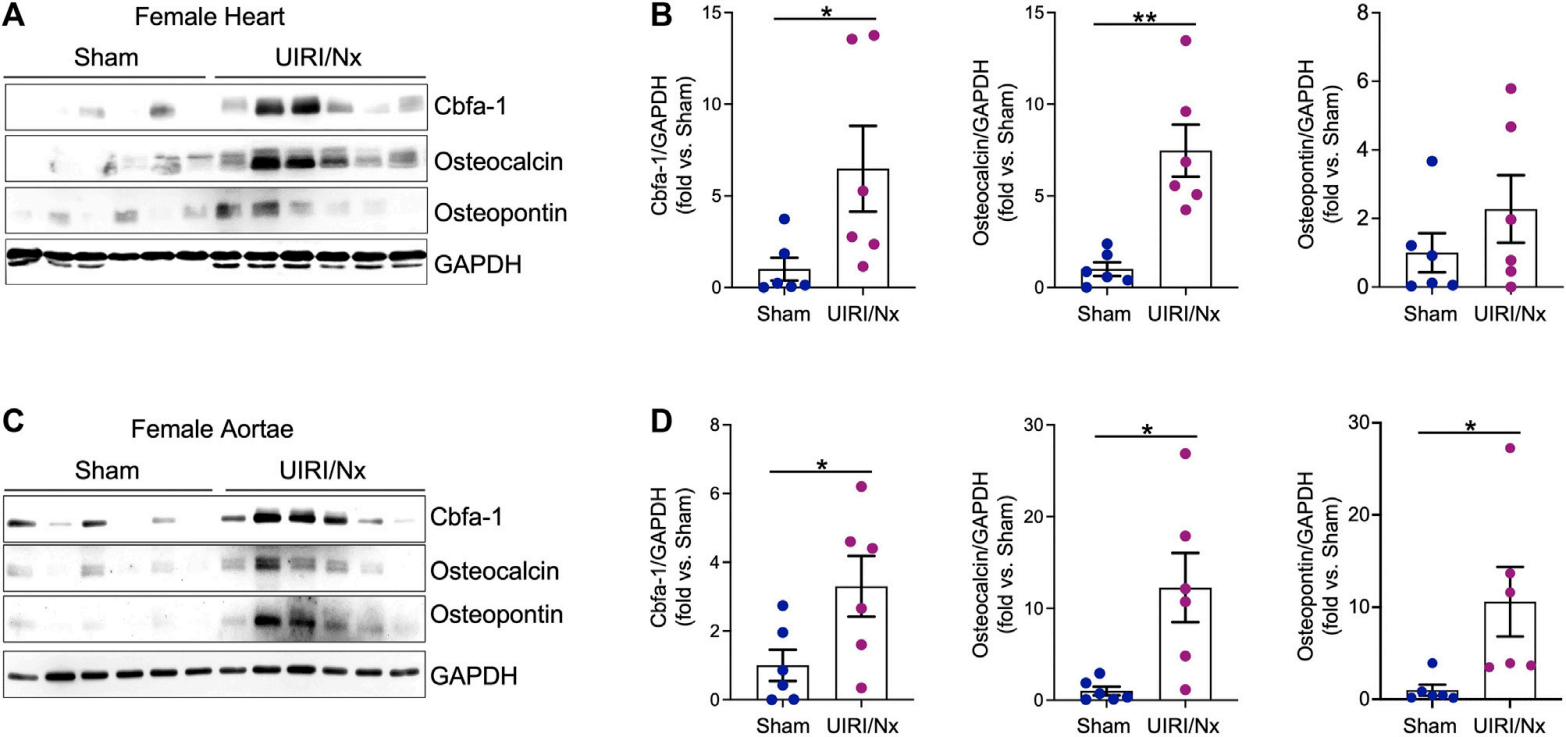
Osteoblastic transition of cardiac tissue and aortae following UIRI/Nx and high dietary phosphate in female mice. **(A)** Protein expression levels of Cbfa-1, osteocalcin, and osteopontin in female cardiac tissues was confirmed by western blot. Blots were stripped and probed for GAPDH. **(B)** Expression of the indicated proteins in the cardiac tissues was analyzed by densitometry, normalized to GAPDH and expressed as fold vs Sham. Data illustrates mean ± SEM. *p < 0.05 vs. Sham, SEM; **p < 0.01 vs. Sham. n = 6/group. **(C)** Western blot depicts protein expression of Cbfa-1, osteocalcin, and osteopontin in female aortae. GAPDH was used as loading control. **(D)** Expression of the indicated proteins in the aortic tissues was analyzed by densitometry, normalized to GAPDH and expressed as fold vs Sham. Data illustrates mean ± SEM. *p < 0.05 vs. Sham. n = 6/group.

**FIGURE 5:**
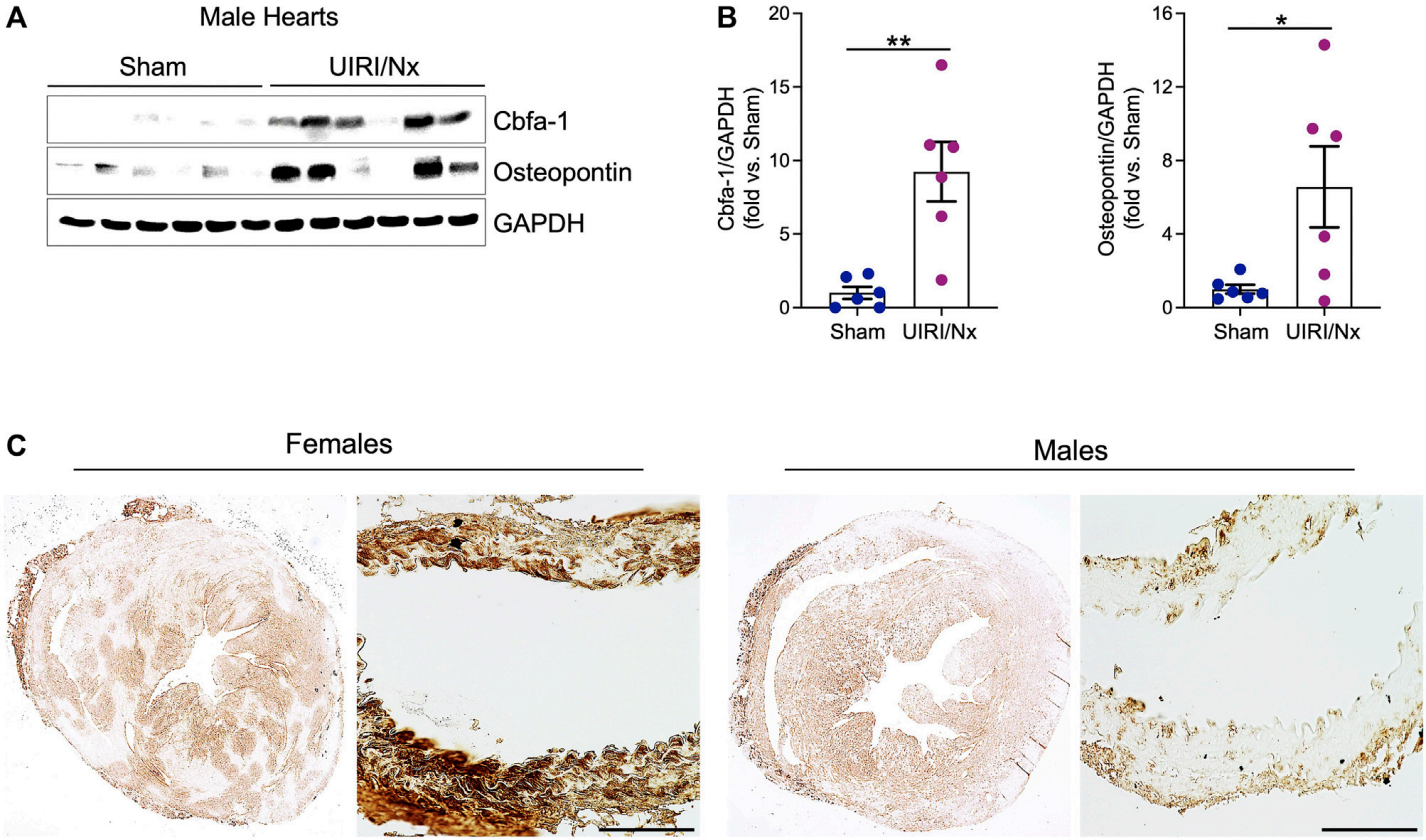
Osteoblastic transition of cardiac tissue following UIRI/Nx and high dietary phosphate in male mice. **(A)** Western blot demonstrates the expression of Cbfa-1, and osteopontin in male cardiac tissue. **(B)** These results were normalized to GAPDH expression and analyzed via densitometry and data expressed as mean ± SEM. Data illustrates fold change vs sham. *p < 0.05 vs. Sham. n = 6/group **(C)** Immunohistochemistry illustrates marked upregulation of osterix in myocardium of both males and females at 16 weeks post UIRI/Nx. Such expression was minimal in aortae of male mice while highly upregulated in aortae of female mice. Scale bar = 100 μm.

CKD associated soft tissue mineralization follows a phenotype switch where SMCs, valvular cells and cardiomyocytes transition into osteoblast like cells and upregulate expression of proteins that are generally expressed by osteoblasts (Zarjou et al., 2009; Nagy et al., 2019; Sikura et al., 2019). We examined level of expression of three proteins that are involved in such osteoblastic transition. Core binding factor-alpha (Cbfa-1) is a key transcription factor that plays a fundamental role in bone formation and osteoblast differentiation, while both osteopontin and osteocalcin are commonly expressed in bones (Ducy, 2000; Zarjou et al., 2010). As illustrated in Figure 4A,B cardiac expression of these osteoblastic markers were significantly higher in female UIRI/Nx hearts compared to sham operated littermates. We also found a similar pattern when we examined the expression level of these proteins in aortae of female mice (Figures 4C,D). To account for gender differences and to further corroborate these findings we also examined the expression levels of aforementioned proteins in cardiac and aortae lysates of male animals (Figure 5A,B). The expression levels of Cbfa-1 and osteopontin were significantly higher following UIRI/Nx in cardiac lysates of male animals. However, we did not detect any osteocalcin in cardiac lysates and none of these markers were found in aortae lysates. To validate these findings, we also performed immunohistochemistry on cardiac and aortae sections of mice that underwent UIRI/Nx to evaluate expression levels of another classic osteogenic marker, namely osterix. As demonstrated in Figure 5C, and in agreement with our western blot analysis, we found prominent expression of osterix in hearts of both males and females. In contrast, our results indicate that such expression was minimal in aortae of male mice but unambiguously upregulated in aortae of female mice (Figure 5C). These findings validate previous reports that female mice are more susceptible to VC, albeit mechanisms behind such differences are yet to be fully elucidated (Qiao et al., 1995).

**FIGURE 6:**
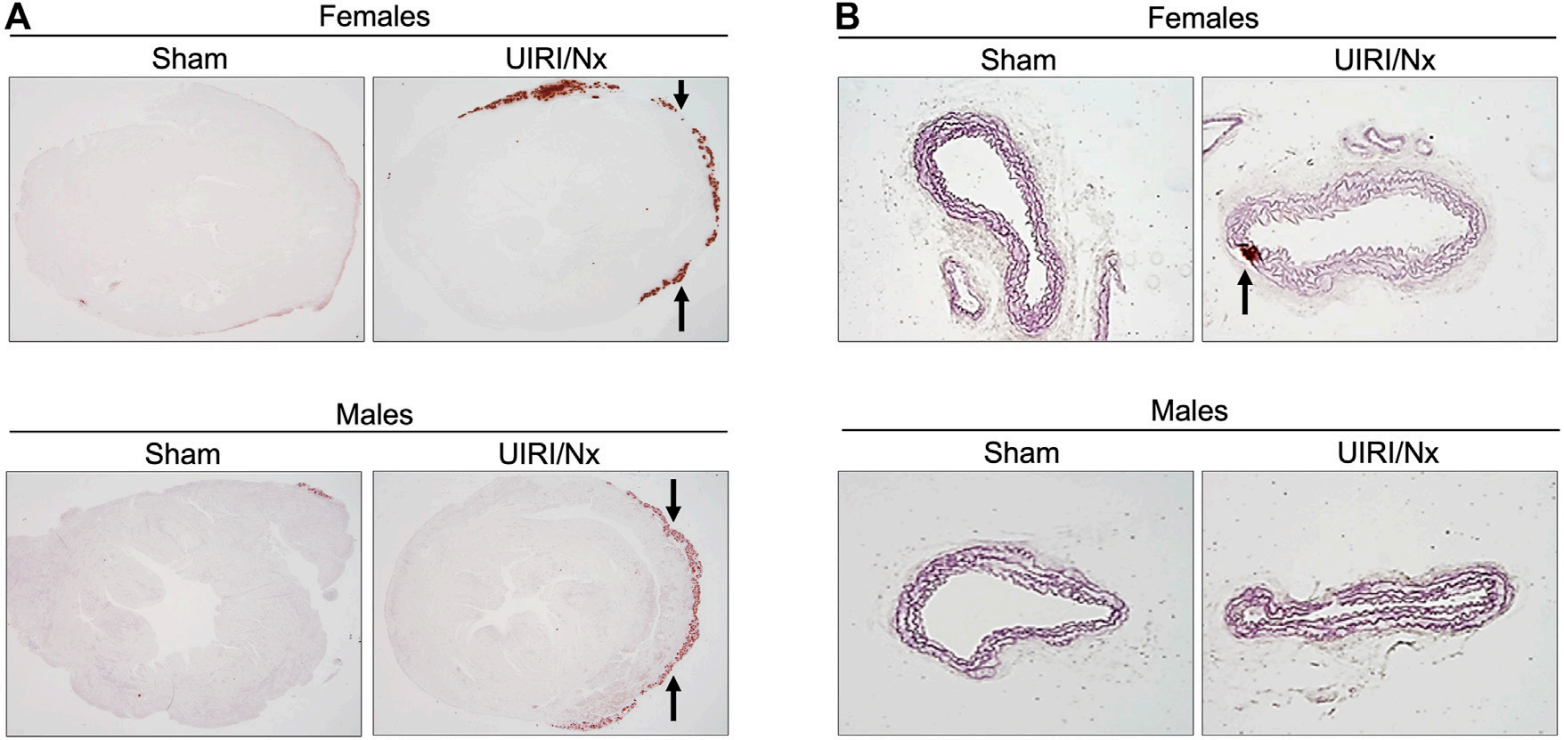
UIRI/Nx model leads to epicardial calcinosis in both genders while aortae reveal scan amount of mineralization only in female aortae. **(A)** Alizarin red stain was used to visualize degree of calcium deposition in cardiac tissues of females (upper panel) and males (lower panel) under sham and experimental condition. Arrows point towards the calcium deposition at the epicardial region. **(B)** abdominal aortae were sectioned and stained with alizarin red. Arrows indicates calcium deposition that was only evident in female aortae. All mice were maintained on 0.9% phosphate and 0.6% calcium for 16 weeks. Images are representative of six independent experiments. Magnification = 4X.

It is well established that DBA/2 mice are not only prone to VC, but also other soft tissues such as kidneys, tongue and myocardium are also prone to calcification (van den Broek et al., 1998). As demonstrated in Figure 6A, cardiac sections of mice that underwent sham surgeries and stained with alizarin red did not manifest any calcium deposition despite higher dietary phosphate content. In contrast, we found a distinct layer of calcium deposition at the level of epicardium in both males and females that underwent UIRI/Nx surgeries (Figure 6A) which further reiterates the need for the microenvironment that is generated by CKD to accelerate the process of mineralization. Furthermore, aortic sections only revealed scant and sporadic amount of calcium deposition at the level of tunica media in female mice that belonged to UIRI/Nx group highlighting early stages of osteoblastic differentiation without overt calcium deposition (Figure 6B). Notably, no such calcification was observed in sham operated animals (Figure 6A,B).

## Discussion

The principal challenge of addressing a clinical condition commences with introduction of relevant animal models. In this study we sought to examine the feasibility and reproducibility of a UIRI/Nx model to induce CKD and its complications including osteoblastic transition of cardiovascular system. Our main emphasis was to ensure that this model would replicate the most prominent complications of CKD in both genders. We demonstrate that mice, irrespective of gender, show similar degree of CKD as evidenced by significant rise in serum creatinine, significant albuminuria, higher degree of collagen deposition, elevated expression of classic fibrotic markers, higher circulating levels of PTH and hepcidin. Moreover, we confirm the osteoblastic transition of SMCs and cardiomyocytes based on higher levels of osteoblastic markers namely, Cbfa-1, osteopontin and osteocalcin. Overall, we found that the significant rise in serum creatinine and albuminuria were modest and did not result in any mortality during the 16 weeks observation period. Additionally, elevated serum hepcidin and PTH, consistent epicardial calcium deposition, upregulation of osteoblastic markers, concomitant with normal levels of serum phosphate and iron further establish the current model as a moderate form of CKD and its related complication. Hence, the presented model may be considered practical for studying: 1) long-term outcomes of CKD, 2) efficacy of early interventions to mitigate CKD progression and its complications. Taken together, our findings demonstrate a reproducible model of moderate CKD with several complications that are commonly associated with early-stage CKD.

Staggering numbers behind the prevalence of CKD and its associated morbidity, mortality, and utilization of health care resources underscore the unmet need for expansion of both preclinical and clinical investigations to alleviate the burden of CKD. This urgent need is also recognized by AAKH initiative (U.S. Department of Health and Human Services, 2019). An animal model that could replicate the pertinent clinical findings utilizing a reasonable amount of time and resources is a requisite for studying any disease condition in preclinical settings. Given the vastly heterogenous nature of CKD and its causes, diversity of animal models to study and generate novel therapeutics that pertain to enhancing the understanding of this devastating disease and discovering novel therapeutics is paramount. However, many mouse models of CKD fail to establish complications related to this disease, particularly as it pertains to sex-differences (Kher et al., 2005; Ramesh and Ranganathan, 2014). This significant challenge that negatively impacts meaningful translational efforts has been well recognized by NIH (Bairey Merz et al., 2019). Therefore, the great emphasis and focus of our study was primarily on developing a consistent model of CKD that would mimic various aspects of this disease commonly observed in humans in both female and male mice. Notably, we demonstrate that this model represents a modest increase in both serum creatinine and degree of albuminuria that would be in line with patients who suffer from CKD stage IIIa-IIIb. Our findings that reveal significant albuminuria, elevation of serum PTH and hepcidin concomitant with normal levels of serum phosphate and iron also substantiate this premise as derangements in serum iron and phosphate levels are only seen in advanced CKD (CKD IV, CKD V, and ESKD) (Zaritsky et al., 2009; Lee et al., 2017).

The frequency of VC in patients with ESKD is striking and nearly half of these patients have valvular mineralization as well (Hujairi et al., 2004; Wang et al., 2018). Mineralization of the vascular tree results in arterial stiffness, with ensuing development or worsening of hypertension and increased pulse wave velocity. Consequently, patients with CKD associated VC are at high risk of left ventricular hypertrophy, coronary and cerebral hypoperfusion and eventual heart failure (Ohishi et al., 2011; Briet et al., 2012; Moody et al., 2013). Accordingly, our study sought to examine degree of such cardiovascular calcification and osteoblastic transition in this moderate CKD model. We demonstrate small amounts of hydroxyapatite calcium deposition in aortae of females who underwent UIRI/Nx, while epicardial calcium deposition was evident in both males and females in the experimental group. Overall, we put more emphasis on female mice given previous evidence that females are more susceptible to VC and our findings corroborate this premise (Qiao et al., 1995; El-Abbadi et al., 2009). Our findings suggest that the process of osteoblastic transition of cardiovascular system starts relatively early in the course of CKD, preceding complications such as hyperphosphatemia and iron deficiency.

We also reason that the level of cardiac calcinosis and medial calcification in this model can be markedly augmented in a shorter observation time allowing for studying varying degrees of VC and related strategies to prevent and slow this process. This concept was shaped via adjusting dietary phosphate levels in a commonly used model of CKD that is well established in our laboratory, namely adenine induced CKD. Here, in agreement with previous reports we found that mice that were fed 0.2% adenine to induce CKD and 2% phosphate had substantially higher degree of calcium deposition in both aortae and myocardium as evidenced by Alizarin red findings illustrated in Supplemental Figure S1. This severe degree of mineralization was present at 4 weeks post commencement of dietary changes. Therefore, increasing dietary phosphate in UIRI/Nx model may also significantly shorten the observation time, and increase the process of calcification, enabling a suitable strategy to examine various aspects of molecular dynamics of this transition and targeting pathways to prevent initiation and propagation of VC.

Compared to rats, induction of CKD in mice is more challenging that stems from multiple factors, (Massy et al., 2007; Shobeiri et al., 2010). The 5/6 nephrectomy model that is overall a consistent and reproducible model in rats, is particularly challenging in mice due to surgical difficulties and inconsistent outcomes (Massy et al., 2007; Shobeiri et al., 2010; Tan et al., 2019). Furthermore, very little renal parenchyma is available at endpoint for investigative purposes. Other utilized models each have their advantages and disadvantages (Massy et al., 2007; Shobeiri et al., 2010). We and others have shown that while bilateral renal IR can be utilized to induce significant AKI, serum creatinine levels mostly return to normal within 4 weeks after surgery (Wei and Dong, 2012; Peng et al., 2015; Zarjou et al., 2019). While this challenge could be addressed by using different ischemia times, the high variance and inconsistencies remain as significant obstacles where too severe AKI leads to marked mortality, while mice with mild AKI fully recover (Fu et al., 2018). Furthermore, female mice are particularly resistant to CKD development in this model further hindering potential opportunities for studying sex differences (Aufhauser et al., 2016). Another commonly used method to induce acute kidney injury and CKD in rodents is cisplatin mediated nephrotoxicity (Perse and Veceric-Haler, 2018; Shi et al., 2018). Similar to other models, female animals are significantly protected against the nephrotoxic effects of cisplatin that makes it difficult to draw generalized conclusions pertaining to pathophysiologic processes and interventional approaches (Perse and Veceric-Haler, 2018). Undoubtedly, the relatively young cohort of animals that are healthy and don’t have major underlying conditions contributes to the aforementioned phenomenon as well. The UIR model has been utilized to study different aspects of AKI, AKI to CKD and CKD pathophysiology (Zager et al., 2013; Polichnowski et al., 2020; Tod et al., 2020). Here, we built on previous experience combining decreased renal mass by performing unilateral nephrectomy and inducing the cascade of AKI to CKD by employing IR. To the best of our knowledge this is the first description of this model within the context of CKD and its associated complications.

Overall, our data suggests that the UIRI/Nx model reliably leads to development of moderate CKD and its related complications through implementing kidney injury in one kidney, and reducing kidney mass by surgical removal of the contralateral kidney. The overall consistency among male and female mice is a prominent strength of this model. However, surgical skills required, and relatively long period of observation could be considered potential pitfalls of this model. Given the vastly heterogenous nature of insults and states of CKD, we reason that addition of this model to the existing models of CKD and associated VC would benefit the target community and allow for more diverse settings to study these clinically germane conditions.

In conclusion, we present a feasible and reproducible model of moderate CKD with associated complications. This premise is supported by markers that are commonly used to define and stage CKD such as, elevated serum creatinine, albuminuria and histological evidence of fibrosis and upregulation of fibrotic markers. Higher levels of circulating hepcidin, FGF-23, and PTH concomitant with preserved levels of serum phosphorus and iron further validate the moderate nature of this CKD model. Additionally, our analysis of the osteoblastic transition of cardiovascular cells despite minimal amounts of calcium deposition highlight the relevance of this model for studying various pathological pathways and potential therapeutic approaches to alleviate the burden of cardiovascular calcification associated with CKD.

## Data Availability

The original contributions presented in the study are included in the article/Supplementary Material, further inquiries can be directed to the corresponding author.

## Supplementary Material

Supplementary Figure S1 Increasing dietary phosphate substantially accelerates development of cardiovascular calcification.

## Abbreviations

AAKH, Advancing American Kidney Health; AKI, acute kidney injury; CKD, chronic kidney disease; cbfa-1, core binding factor subunit alpha-1; ELISA, enzyme-linked immunoassay; ESKD, end stage kidney disease; FGF-23, fibroblast growth factor-23; GAPDH, glyceraldehyde 3-phosphate dehydrogenase; HRP, horseradish peroxidase; PBS, phosphate buffered saline; PSR, picrosirius red; PTH, parathyroid hormone; PVDF, polyvinylidene difluoride; SMCs, smooth muscle cells; SEM, standard error of measurement; UIRI/Nx, unilateral ischemia-reperfusion model followed by contralateral nephrectomy; VC, vascular calcification.
