# Supplementary figures and images for: A Reproducible Mouse Model of Moderate CKD With Early Manifestations of Osteoblastic Transition of Cardiovascular System
Source: Front Physiol. 2022 Apr 29;13:897179. doi: 10.3389/fphys.2022.897179 (PMC9099146; doi:10.3389/fphys.2022.897179)

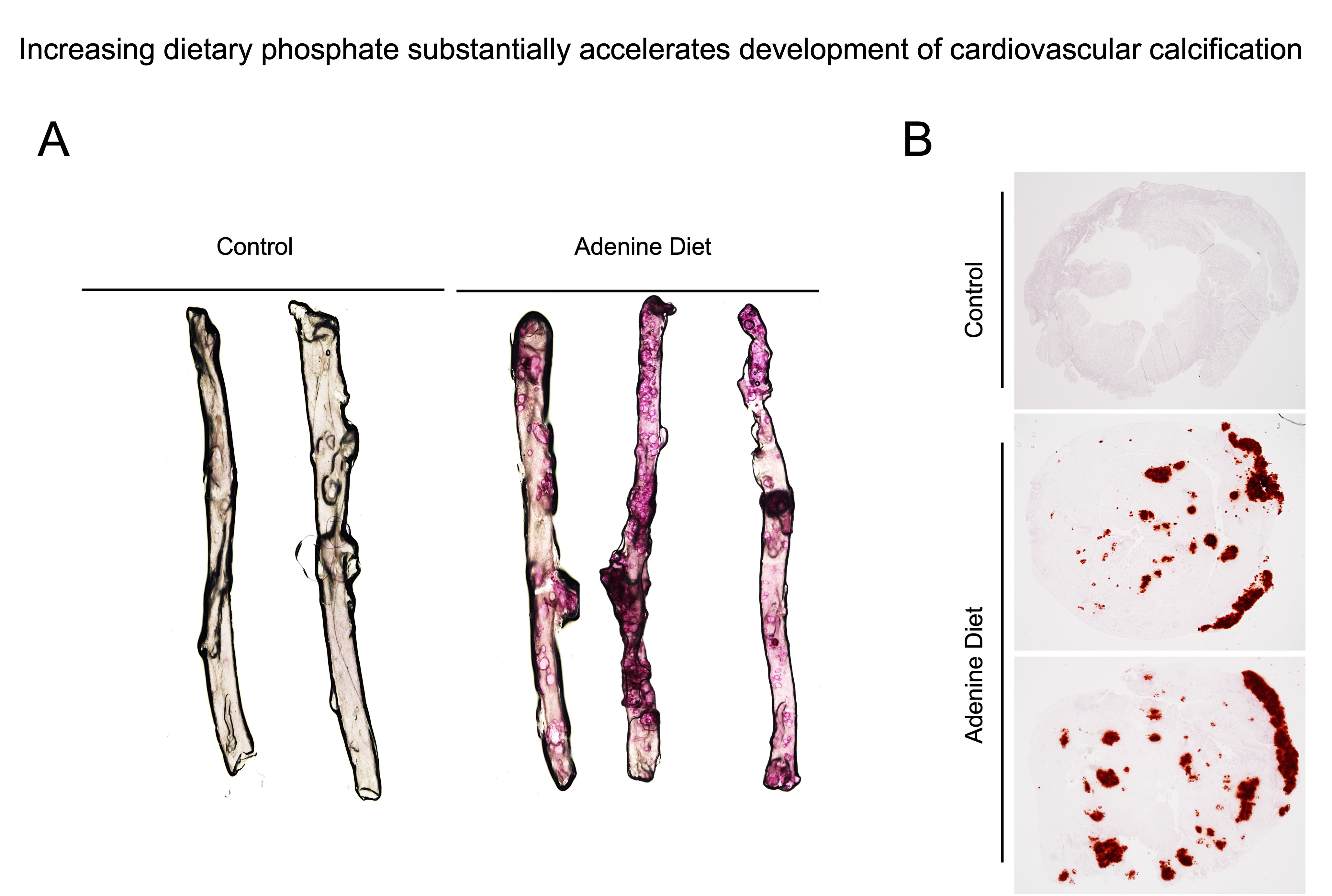

Supplement: Supplementary file 1 [file Image1.jpg]
